# Effect of hydroxyurea on the promoter occupancy profiles of tumor suppressor p53 and p73

**DOI:** 10.1186/1741-7007-7-35

**Published:** 2009-06-26

**Authors:** Vera Huang, Xin Lu, Yong Jiang, Jean YJ Wang

**Affiliations:** 1 Division of Biological Sciences, University of California, San Diego, La Jolla, CA 92093-0820, USA; 2 Division of Hematology-Oncology, Department of Medicine, School of Medicine, University of California, San Diego, La Jolla, CA 92093-0820, USA; 3 Moores Cancer Center, University of California, San Diego, La Jolla, CA 92093-0820, USA; 4 Department of Family and Preventive Medicine, Division of Bioinformatics and Biostatistics, University of California, San Diego, La Jolla, CA 92093, USA; 5 Current address: Department of Urology and Helen-Diller Family Comprehensive Cancer Center, University of California, San Francisco, San Francisco, CA 94158, USA; 6 Current address: Abbott Laboratories, 100 Abbott Park Road, North Chicago, IL 60063, USA; 7 Current address: Wintherix LLC, 10578 Science Center Drive, CB-7, Ste 1133, San Diego, CA 92121, USA

## Abstract

**Background:**

The p53 tumor suppressor and its related protein, p73, share a homologous DNA binding domain, and mouse genetics studies have suggested that they have overlapping as well as distinct biological functions. Both p53 and p73 are activated by genotoxic stress to regulate an array of cellular responses. Previous studies have suggested that p53 and p73 independently activate the cellular apoptotic program in response to cytotoxic drugs. The goal of this study was to compare the promoter-binding activity of p53 and p73 at steady state and after genotoxic stress induced by hydroxyurea.

**Results:**

We employed chromatin immunoprecipitation, the NimbleGen promoter arrays and a model-based algorithm for promoter arrays to identify promoter sequences enriched in anti-p53 or anti-p73 immunoprecipitates, either before or after treatment with hydroxyurea, which increased the expression of both p53 and p73 in the human colon cancer cell line HCT116-3(6). We calculated a model-based algorithm for promoter array score for each promoter and found a significant correlation between the promoter occupancy profiles of p53 and p73. We also found that after hydroxyurea treatment, the p53-bound promoters were still bound by p73, but p73 became associated with additional promoters that that did not bind p53. In particular, we showed that hydroxyurea induces the binding of p73 but not p53 to the promoter of *MLH3*, which encodes a mismatch repair protein, and causes an up-regulation of the *MLH3 *mRNA.

**Conclusion:**

These results suggest that hydroxyurea exerts differential effects on the promoter-binding functions of p53 and p73 and illustrate the power of model-based algorithm for promoter array in the analyses of promoter occupancy profiles of highly homologous transcription factors.

## Background

The p53-family of transcription factors, p53, p63, and p73, regulate genes involved in DNA repair, cell cycle checkpoints, and apoptosis in response to cellular stress [1]. Mouse genetics studies have suggested that these transcription factors have common and unique biological functions. In contrast to p53-deficient mice, which are predisposed to early cancer development [2], mice with loss of p63 or p73 have profound defects in their epithelial and neuronal development, respectively [3,4]. Compound heterozygous p63^+/-^p53^+/- ^or p73^+/-^p53^+/- ^mice were found to have higher incidence of tumorigenesis and increased metastatic ability than p53^+/- ^single heterozygous mice, suggesting a collaborative role for the p53-family in tumor suppression [5]. In addition, the combined loss of p73 and p53 induces genomic instability more severely than that induced by loss of p53 alone [6]. Taken together, these observations suggest that p53, p63, and p73 have redundant as well as non-overlapping functions.

With the advent of the chromatin immunoprecipitation on DNA chip (ChIP-chip), which allows for a genome-wide analysis of transcription factor-binding sites in cells, a number of studies have been conducted to identify genomic-binding sites of the p53-family members [7-14]. These studies have each analyzed the binding sites of an individual member of the p53-family, notably p53 itself. In one study, a comparison of the genomic-binding sites between p53 and p73 was carried out under conditions of over-expression [14]. In this study, we used the NimbleGen 1.5-kb promoter array platform covering 24,135 human promoters to examine the promoter occupancy profiles of endogenous p53 and p73 in the human colon cancer line HCT116-3(6), both before and after hydroxyurea (HU) treatment. We developed a model-based analysis of the hybridization results and identified a series of p53 and p73 associated promoters. This study has revealed a previously unknown effect of HU on the promoter occupancy profiles of two highly related transcription factors.

## Results

### Establishing the experimental system

**Figure 1:**
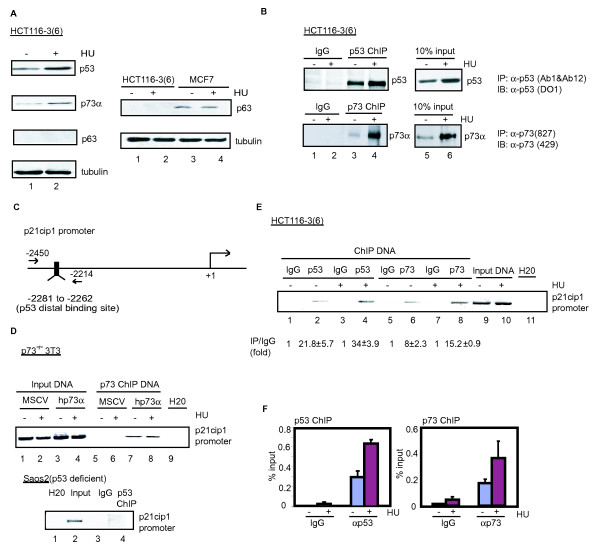
**Hydroxyurea-induced accumulation of p53 and p73 and increased occupancy of the p21cip1 promoter in HCT116-3(6) cells**. (a) The indicated cells were treated with or without HU (1 mM) for 16 hours, and the indicated proteins were detected by immunoblotting as described in Methods. (b) Higher levels of p53 and p73 induced by HU correlates with increased association with chromatin. HCT116-3(6) cells with or without HU treatment were subjected to chromatin immunoprecipitation (ChIP) using the indicated antibodies followed by immunoblotting with the indicated antibodies. (c) Polymerase chain reaction (PCR) primer designed for the distal p53 binding site in the p21cip1 promoter. (d) ChIP specificity. p73 -/- 3T3 cells reconstituted with either the empty vector or human p73α were treated with or without HU as in (a) and were subjected to ChIP using the indicated antibodies. Enrichment of the p21cip1 promoters was assessed by PCR using primers encompassing the p53-binding site in the distal region of the p21cip1 promoter as shown in (c). (e) Increased occupancy of the p21cip1 promoter by p53 and p73 following HU treatment. Chromatin from HCT116-3(6) cells with or without HU treatment were immunoprecipitated with the indicated antibodies (lanes 2, 4, 6, and 8) and followed by PCR analysis using the primers as shown in (c). M = mouse immunoglobulin G (IgG); R = rabbit IgG. Fold enrichment relative to the IgG sample was determined by quantitative PCR (f) Quantitative real time-PCR analysis of (e) shown as percent total input DNA.

The colon cancer cell line HCT116-3(6) expresses p53 and p73 at a much higher level than p63, shown by immunoblotting (Figure 1a, left panel) and quantitation of mRNA (see Figure S1A in Additional file 1); furthermore, treatment with HU increased the steady-state levels of p53 and p73, but not p63 (Figure 1a, left panel). The p63 protein was detectable in MCF7 cells, but HU did not increase its level in this breast cancer cell line (Figure 1a, right panel). Time-course experiments showed that p53 levels increased steadily between 12 hours and 48 hours after HU addition, while p73 levels reached a peak at 24 hours of HU addition (see Figure S1B in Additional file 1). We therefore performed subsequent experiments with a 16-hour treatment of HU, a time at which both p53 and p73 levels were higher than the basal levels.

We prepared affinity-purified anti-p73 polyclonal antibody and demonstrated that the anti-p73 (827) antibody reacted with four isoforms of p73 (α, β, γ and δ) but not p53 (see Figure S1C in Additional file 1). We performed chromatin immunoprecipitations (ChIPs) with anti-p53 and our home-made anti-p73 (827) antibodies followed by Western blotting and observed higher levels of p53 and p73 in cross-linked chromatin preparations from HU-treated cells (Figure 1b), consistent with increased levels of both proteins (Figure 1a). Therefore, the antibodies can react with p53 or p73 and HU treatment did not appear to mask the p53 and p73 epitopes in the context of cross-linked chromatin.

We established the specificity of anti-p53 and anti-p73 ChIP, taking advantage of the fact that p53 and p73 both bind to the distal p53 response element located about 2.3 kb upstream of the transcription start site in the p21cip1 promoter [15,16] (Figure 1c). To test the specificity of our anti-p73 (827) antibody, we performed ChIP in p73^-/- ^3T3 cells reconstituted with either the empty murine stem cell virus (MSCV) vector or human p73-α. We found that our anti-p73 antibody specifically brought down the p21cip1 promoter-distal region in p73^-/- ^3T3 cells reconstituted with human p73-α but not with MSCV (Figure 1d, top panel). Similarly, we performed ChIP with anti-p53 in the p53-deficient human Saos2 cells and detected no enrichment of the p21cip1 promoter-distal region (Figure 1d, bottom panel). Next, we used cross-linked chromatin from untreated and HU-treated HCT116-3(6) cells to perform ChIPs with antibodies against p53 or p73 and measured the enrichment of the p21cip1 promoter sequence in the immunoprecipitates by quantitative polymerase chain reaction (qChIP). We found that both p53 and p73 occupied the distal binding site in the p21cip1 promoter (Figure 1e). Moreover, p21cip1 promoter occupancy by p53 and p73 was increased following HU treatment (Figure 1e and 1f). Taken together, these results showed that the HCT116-3(6) cells and the ChIP conditions we established can be used to study the promoter occupancy profiles of p53 and p73.

### ChIP-chip experiments using promoter arrays

**Figure 2:**
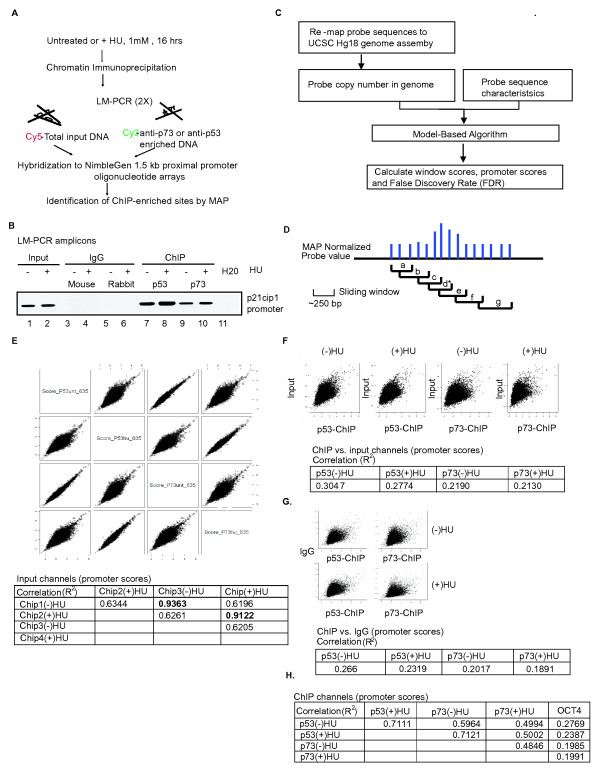
**Model-based analysis for promoter array**. **(a) **Scheme for chromatin immunoprecipitation (ChIP) on DNA chip analysis. **(b) **Confirmation of ChIP and ligation-mediated -polymerase chain reaction amplified amplicons. ChIPs were performed as described in Figure 1(e). Enrichment of the p21cip1 distal promoter region was used to check the specificity of the samples prior to hybridization, M = mouse immunoglobulin (IgG); R = rabbit IgG. **(c) **Outline of the bioinformatics analysis for the identification of p53 and p73 ChIP-enriched promoters. **(d) **Definition of window and promoter-scores. a-g: window score; *: promoter-score. **(e) **Pair-wise scatter plots of the promoter-scores for the input channels (top). *R*^2 ^values are summarized in the table. **(f) **Pair-wise scatter plots of the promoter-scores between the ChIP and input channels (top). *R*^2 ^values are summarized in the table. **(g) **Pair-wise scatter plots of the promoter-scores between the ChIP and IgG channels (top). *R*^2 ^values are summarized in the table. **(h) **Correlation between the ChIP channels and OCT4 ChIP control. *R*^2 ^values are summarized in the table.

Previous studies have shown that the majority of p53 genomic-binding sites fall outside of the promoter region [7,13]. In this study, we used the 1.5-kb NimbleGen promoter arrays, which contain probes corresponding to the -1.3-kb to +200-bp region relative to the transcription start site of 24,135 human genes, and thus limiting the comparison only to the promoter occupancy profiles of p53 and p73. To generate the hybridization probes, cross-linked chromatin from untreated or HU-treated HCT116-3(6) cells were immunoprecipitated with anti-p53 or anti-p73 and the resulting DNA was amplified by ligation-mediated PCR (LM-PCR) (Figure 2a). The specificity of the amplified DNA was confirmed by PCR using primers specific for the p21cip1 promoter-distal region (Figure 2b). As expected, the p21cip1 promoter-distal region was enriched in the amplicons derived from the anti-p53 and anti-p73 ChIP, but not from the immunoglobulin G (IgG) ChIP (Figure 2b). We then labeled the p53-ChIP or p73-ChIP amplicons and the total input amplicons with Cy3 and Cy5 dye, respectively, and co-hybridized input and ChIP DNA probes from the -HU or +HU condition to four NimbleGen 1.5-kb promoter arrays (Figure 2a).

We developed a model-based algorithm for promoter arrays (MAP), which is similar to the model-based algorithm for tiling arrays (MAT) [17] to analyze the hybridization results (Figure 2c and details can be found in Additional data file 2). The NimbleGen promoter array platform used in this study contains 15 50-mer probes tiled across each 1.5-kb promoter-proximal region. We applied MAP to obtain standardized fluorescence values for each probe on the array, following adjustments for probe copy numbers and guanine-cytosine content [17]. We then calculated a series of window scores, which are the median of the MAP values of three probes within a series of sliding windows (Figure 2d). The highest window score is then assigned as the promoter-score (Figure 2d). A false discovery rate (FDR) is calculated for each promoter-score according to the method developed by Storey and Tibshirani [18]. Instead of taking the ratios of promoter-scores between the input and ChIP DNA, we compared the promoter-scores and the FDR of the input and ChIP channels as independent entities (see Additional file 3).

We first compared the promoter-scores deduced from hybridization with the total input DNA (Figure 2e), and found the *R*^2 ^value of 0.9363 (-HU) and 0.9122 (+HU) between two independent hybridizations, demonstrating a high degree of technical reproducibility (Figure 2e). The *R*^2 ^values between input DNA from -HU versus +HU cells were in the range 0.6196 to 0.6344, representing the experimental correlation generated from two different biological samples (Figure 2e). Comparison of input versus ChIP signals on each of the four arrays showed *R*^2 ^values in the range 0.2130 to 0.3047 (Figure 2f). We then calculated the MAP scores from an IgG-ChIP DNA hybridization result from Xu *et al*. [19], and found similarly low *R*^2 ^values in the range 0.1891 to 0.2660 when compared with the p53- or p73-ChIP DNA (Figure 2g). In addition, we calculated the MAP scores from an Oct4-ChIP DNA result from Jin *et al*. [20] and found *R*^2 ^values again to be in the range 0.1991 to 0.2760 when compared with the p53- or p73-ChIP DNA (Figure 2h). It should be noted that the MAP scores deduced for input DNA from different laboratories correlated with one another at *R*^2 ^values of 0.5, which was in the range (i.e. 0.6) of the two input DNA comparisons from this study. These results demonstrate the feasibility of using MAP to compare the different hybridization datasets obtained from the same array platform. When the comparisons were made between p53- and p73-ChIP DNA, we found that the *R*^2 ^values were in the range 0.4846 to 0.7121 (Figure 2h), which were in the range of comparing input DNA hybridization results (biological repeats) from different laboratories. Taken together, these results indicated that p53- and p73-ChIP DNA shared a high degree of similarity, which was significantly different from the total input DNA and the IgG- and Oct4-ChIP DNA.

### Validation of the MAP window and promoter-scores

To determine experimentally the confidence level of the MAP-calculated scores, we performed qChIP on a series of promoters selected across MAP-deduced FDR values from 0 to 0.05 (see Figure S2 in Additional file 1). We calculated occupancy units (OU) from each qChIP experiment as previously described in Yang *et al*. [8]. We found that the log2 OU for the negative controls fell significantly below 1 (*P *< 0.005), thus, we assigned log2 OU of >1 as a positive qChIP result, indicative of p53 or p73 binding to a promoter region. We scanned the 1.5-kb region of an X-chromosome promoter identified by MAP to bind p53 and p73 by qChIP, and found that the log2 OU correlated well with the MAP-deduced window scores (see Figure S2a in Additional file 1). We then performed qChIP on 20 selected promoters using primers flanking each of the 250 bp windows with the peak MAP scores (see Figure S2b in Additional file 1). We chose these 20 promoters for qChIP verification experiments because they showed no significant enrichment in the input DNA samples (FDR > 0.8), but in the four ChIP DNA samples (p53+/-HU, p73+/-HU, FDR < 0.05). The results of qChIP OU values of these 20 promoters from each of the four experimental conditions (p53-ChIP +/- HU, p73-ChIP +/- HU) are summarized in Figure S2c in Additional file 1. We also performed qChIP with IgG, and confirmed that there was no significant enrichment of these 20 promoters in the IgG controls (data not shown).

Based on the qChIP results of the 20 selected promoters, we determined the false discovery rate (FDR_qChIP_) using a weighted approach [8]. We separated the FDR_MAP _values into three bins (0 to 0.005, 0.005 to 0.01, 0.01 to 0.05) (see Figure S2d in Additional file 1) and assigned FDR_qChIP _to each bin (see Figure S2d in Additional file 1). At FDR_MAP _< 0.005, the FDR_qChIP _was 17% for p53 (-HU), suggesting a confidence level of 83%. Similarly, at FDR_MAP _< 0.005, the FDR_qChIP _was 21% for p73 (+HU), with a confidence level of 80% (see Figure S2d in Additional file 1). We then counted the number of promoters showing FDR_MAP _< 0.005 from each of the four ChIP hybridizations and FDR_MAP _> 0.8 in the corresponding input hybridization (for a complete list of these promoters, refer to Additional file 4). The number of promoters identified this way are: 201 for p53(-HU), 216 for p53(+HU), 360 for p73(-HU), and 526 for p73 (+HU) (see Figure S2d in Additional file 1).

### Comparison of p53 and p73 promoter occupancy profiles

**Figure 3:**
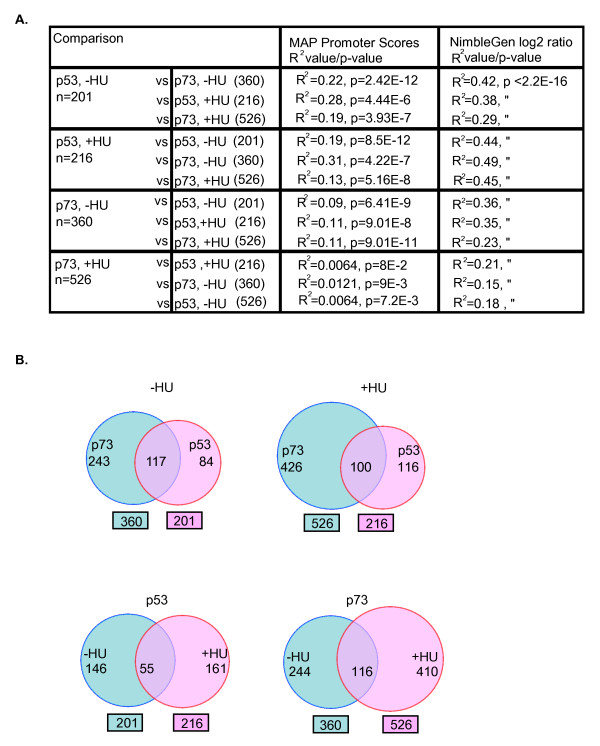
**Comparison of p53 and p73 promoter occupancy profiles**. **(a) **Summary of the correlation analysis on selected promoters using model-based algorithm for promoter arrays promoter-scores (column 3), or NimbleGen log2 ratios (column 4). *R*^2 ^values represent the degree of correlation and *P *values represent the significance of correlation. **(b) **Venn diagrams depicting the number of common and distinct promoters occupied by p53 and/or p73 using FDR_MAP _< 0.005.

We used linear regression to compare the promoter-scores from different experiments, because it introduces the least manipulation (Figure 3). As discussed above, the *R*^2 ^value was 0.5964 when the total promoter-scores from the p53-ChIP (-HU) and the p73-ChIP (-HU) experiments were compared (Figure 2h). We noted that the *R*^2 ^values were consistently lower when comparisons were made between p73-ChIP (+HU) and any of the other three ChIP DNA samples (Figure 2h). After the selection of promoters of confidence levels between 60% and 80% (see Figure S2d in Additional file 1), we compared their MAP-deduced promoter-scores across the four ChIP hybridizations: p53 (-HU), p53 (+HU), p73 (-HU), and p73 (+HU) (Figure 3). As would be expected, the overall correlation was reduced when the p73 (+HU) subset of promoter-scores was compared. We found *R*^2 ^values ranging from 0.09 to 0.28 when the promoter sets were selected from the p53 (-HU), p53 (+HU), and p73 (-HU) hybridization experiments (Figure 3a). However, the *R*^2 ^values were in the range 0.006 to 0.01 with the promoter set selected from the p73 (+HU) hybridization experiment (Figure 3a). The overlaps among the four subsets of promoters are also depicted as Venn diagrams (Figure 3b). Taken together, the correlation analysis suggested that while p53 and p73 bound to similar promoters at steady state and after HU-treatment, p73 appeared to bind to additional promoters in HU-treated cells.

**Figure 4:**
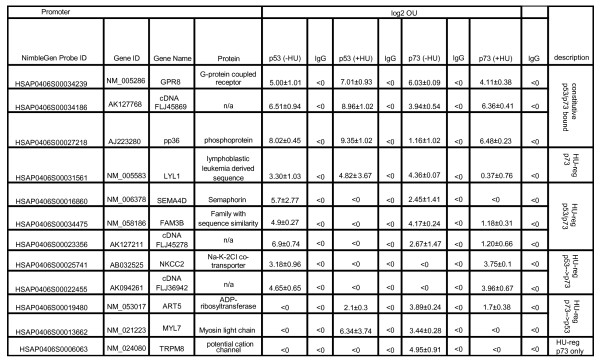
**Summary of quantitative chromatin immunoprecipitation analysis of selected promoters for p53 and p73 binding before and after hydroxyurea treatment**. Cross-linked chromatin from untreated hydroxyurea (-HU) or hydroxyurea-treated (+HU) (1 mM, 16 hours) HCT116-3(6) cells were immunoprecipitated with the indicated antibodies and followed by polymerase chain reaction analysis using the primers flanking the sequences corresponding to the model-based algorithm for promoter array promoter-score window in each of the promoters. Data shown are log2 occupancy units from two independent chromatin immunoprecipitation experiments +/- standard deviation. log2 occupancy units >1: significant enrichment of the selected promoter sequences in the anti-p53 or anti-p73 chromatin immunoprecipitation.

Next, we selected additional binding sites for qChIP verification to identify HU-induced and p73-specific binding sites by the following criteria: those with FDR_MAP _< 0.005 in the p73-ChIP +HU hybridization experiment and FDR_MAP _> 0.8 in the other three ChIP hybridization experiments, and FDR_MAP _> 0.8 in all four input hybridizations. In addition, we selected other promoters with FDR_MAP _< 0.005 in at least one of the four ChIP hybridization experiments and FDR_MAP _> 0.8 in all four input hybridizations (Figure 4). All together, 12 promoters with different binding patterns were tested by qChIP. We verified that p53 and p73 indeed occupied common and distinct promoters identified by MAP. Out of the 12 promoters tested, three were constitutively bound by p53 and p73 in cells untreated or treated with HU (Figure 4). With the other nine promoters, we found HU-regulated binding in several combinations (Figure 4). These qChIP results demonstrate that the MAP-generated data from this study are useful in identifying p53 and/or p73-associated promoters in untreated and HU-treated cells.

### Correlation between promoter occupancy and regulation of gene expression

**Figure 5:**
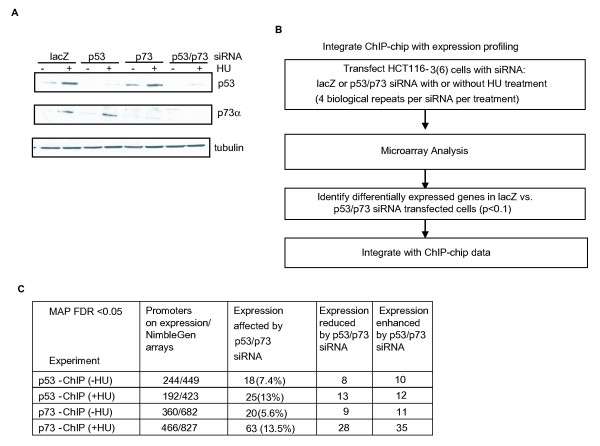
**Integration of chromatin immunoprecipitation on DNA chip and gene expression profiling**. **(a) **Small interfering RNA (siRNA)-mediated knockdown of p53 and/or p73 in HCT16-3(6) cells. Immunoblot of lysates from untreated or hydroxyurea-treated HCT116-3(6) cells (1 mM, 16 hours) following transfection with chemically synthesized siRNA oligos against lacZ, p53, and/or p73. **(b) **Flow chart for the integrated analysis of chromatin immunoprecipitation on DNA chip with expression profiling. **(c) **Summary of the relationship between p53/p73 binding and gene expression. Column 4: expression in p53 and p73 siRNA-transfected cells is lower than that of lacZ-transfected cells. Column 5: expression in p53 and p73 siRNA-transfected cells is higher than that of lacZ-transfected cells.

To examine the relationship between promoter occupancy and gene expression, we depleted p53 and p73 via small interfering RNA (siRNA) in HCT116-3(6) cells and then performed microarray analysis of 32,000 genes before or after HU treatment using the Phalanx expression array platform (Figure 5a and 5b). Genes that are common between the NimbleGen and Phalanx arrays were then analyzed (Figure 5c). We found that between 6% and 14% of p53 and p73 bound promoters at FDR_MAP _< 0.05 exhibited significant changes in mRNA expression when p53 and p73 were simultaneously knocked down by siRNA (Figure 5c). Among them, we identified 18 p53-bound promoters that showed a significant change in gene expression when p53 and p73 were knocked down in the -HU condition (Figure 5c and Additional file 5). Among these 18 promoters, we found that the expression of metalloproteinase 12 (*MMP12*) was up-regulated upon depletion of p53 and p73, consistent with a previous report that p53 is a negative regulator of *MMP9 *[21] (see Additional file 5). We also identified 20 p73-bound promoters that showed a significant change in gene expression when p53 and p73 were simultaneously knocked down under the -HU condition (see Additional file 5). Moreover, we identified 25 p53-bound promoters that showed a significant change in gene expression when p53 and p73 were simultaneously knocked down in the +HU condition (see Additional file 5). Under the same condition, we identified 63 p73-bound promoters whose expression was significantly affected by the double knockdown of p53 and p73 (see Additional file 5). Taken together, while the correlation between binding and regulation of expression is limited [8,22-24], the combination of ChIP-chip and gene expression profiling with siRNA is feasible in the identification of p53/p73-regulated promoters.

### MLH3 as a p73-specific target in HU-treated cells

**Figure 6:**
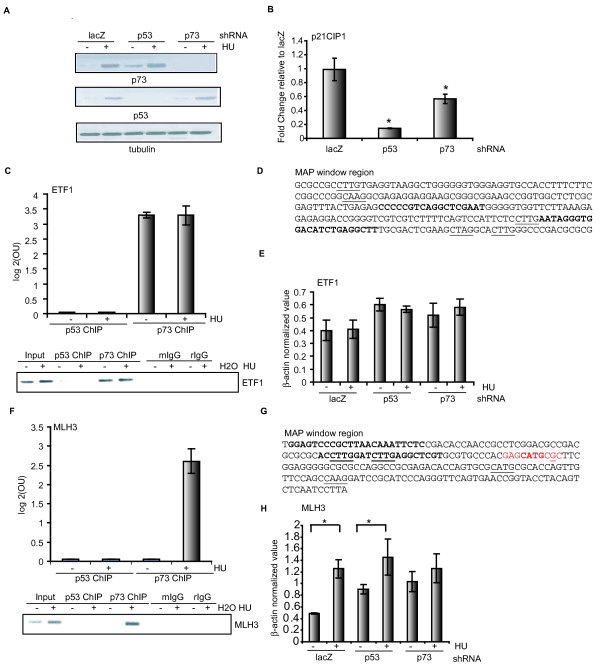
**MLH3 is a p73-specific target induced by hydroxyurea**. **(a) **Western blotting analysis of lysates from untreated or hydroxyurea (HU)-treated HCT116-3(6) cells (1 mM, 16 hours) stably expressing lacZ, p53, or p73 small hairpin RNA (shRNA). **(b) **Effect of p53 or p73 knockdown on p21cip1 gene expression. Real-time RT-PCR analysis of the steady-state p21cip1 mRNA level in HCT116-3(6) cells stably expressing p53 or p73 shRNA *P < 0.05, n = 3 **(c) **Chromatin from untreated or HU-treated HCT116-3(6) cells (1 mM, 16 hours) were immunoprecipitated with the indicated antibodies and followed by PCR analysis using the primers flanking the ETF1 promoter region defined by the model-based algorithm for promoter array (MAP) promoter-score. Data are represented as log2 occupancy units from two independent ChIP experiments +/- standard deviation. Enrichment was confirmed by agarose gel electrophoresis analysis (bottom). **(d) **MAP window sequence. Primers used for qChIP analysis in (c) are indicated in bold. The core consensus sequence of p53 is underlined. **(e) **ETF1 mRNA level in the stable cell lines described in (a) with or without HU treatment was examined by RT-PCR analysis. **(f) **qChIP analysis of the MLH3 promoter was performed as in (c). **(g) **MAP window sequence. Primers used for qChIP analysis in (f) are shown in bold. The core consensus sequence of p53 is underlined. The p53 half site is highlighted in red and underline indicates mismatches. **(h) **Effect of p53 or p73 knockdown on *MLH3 *gene expression. Same analysis was carried out as in (e). *P < 0.05, *n *= 3.

As the correlation analysis suggested that unique promoter sequences were enriched in the p73-ChIP (+HU) experiment, we further examined some of these promoters that are bound by p73 but not p53 in HU-treated cells. By comparing the four selected promoter sets (see Figure S2d in Additional file 1 and Figure 3), the MAP analysis suggested that p73 was selectively bound to 351 promoters in HU-treated cells (see in Additional file 6). Among them, we tested two promoters by qChIP: *MLH3*, a DNA mismatch repair gene involved in microsatellite instability as well as meiotic arrest [25,26], and *ETF1*, eukaryotic translation termination factor gene 1, involved in the termination translation [27]. We performed RT-PCR experiments and measured the mRNA levels in HCT116-3(6) cells stably expressing p53 or p73 small hairpin RNA (shRNA) (Figure 6a). As a positive control, we showed that the knockdown of p53 or p73 each reduced the steady-state levels of p21Cip1 in untreated cells albeit to different extents (Figure 6b). We found that the ETF1 promoter sequence was enriched in the anti-p73 but not the anti-p53 ChIP (Figure 6c). The qChIP experiment showed that p73 binding was detected in untreated and HU-treated cells (Figure 6c). The peak MAP window in the ETF1 promoter contains a p53-consensus binding sequence (Figure 6d), although our current data are not sufficient to support the conclusion that p73 specifically bound to that consensus sequence. HU treatment did not alter the mRNA levels of ETF1, and the knockdown of p53 or p73 also did not affect its expression in response to HU (Figure 6e). Therefore, the association of p73 with ETF1 appeared to be inconsequential to its expression in HCT116-3(6) cells. By contrast, the qChIP results showed that p73 associated with the MLH3 promoter only in HU-treated cells, and that p53 did not bind to this promoter in either untreated or HU-treated cells (Figure 6f). Again, we detected a p53-consensus sequence in the window corresponding to the peak MAP score (Figure 6g). Furthermore, we detected a low but statistically significant induction of MLH3 mRNA in HU-treated cells, with either the LacZ or the p53 shRNA (Figure 6h). Interestingly, the knockdown of p73 abolished the HU-induced up-regulation of MLH3 (Figure 6h), indicating that the binding of p73 to this promoter correlated with an up-regulation of its expression.

## Discussion

In this study, we established an experimental system to examine the *in vivo *promoter occupancy profiles of p53 and p73 before and after HU treatment (Figure 1 and Figure S1 in Additional file 1). Using a model-based analysis (MAP) of array hybridization signals, we identified p53- and p73-ChIP-enriched promoters and determined the qChIP FDRs (Figure 2 and Figure S2 in Additional file 1). Comparisons of promoter sets selected with FDR_MAP _of < 0.005 showed that HU induces the binding of p73 to unique promoters not shared by p53 (Additional files 4 and 6). Gene ontology (GO)-based analyses showed that these HU-induced p73-binding promoters belong to distinct GO terms (Additional file 7). Among them, we have confirmed the MLH3 promoter as a HU-induced and p73-specific target (Figure 6). Our study has gone beyond the simple cataloging of p53 and p73 binding sites and demonstrated the power of MAP in elucidating the promoter-binding functions of p53 and p73 before and after the exposure of cells to genotoxic stress.

### MAP analysis of ChIP-chip results

Rank-based target identification [28] or single-array model [29] are the two main statistical approaches that have been employed to analyze ChIP-chip data. In both methods, the enrichment is defined as the ratio of the ChIP DNA signal over the input DNA signal on the array. In the rank-based target identification method, genomic loci are ranked according to their fluorescence ratios and a median percentile rank for calling a positive target is determined across independent micro-array hybridizations. The single-array error method assumes that the log2 ratios in a ChIP-chip experiment follow a normal distribution and determines the significance of the enrichment by calculating a one-sided probability. Targets are usually selected by a *P *value cutoff. Application of these analytic methods requires multiple replicates that are prohibitively expensive with the ChIP-chip experiments.

Johnson *et al*. [17] developed MAT, which utilizes data from a single chip for standardization without the requirement of multiple replicates by modeling the baseline probe behavior based on probe sequence characteristics and probe copy number in the genome. Adopting the principles of MAT [17], we demonstrated the power of a model-based algorithm for ChIP-chip data analysis on promoter arrays (termed MAP). Our study demonstrates that it is possible to obtain new insights on the promoter occupancy profiles of transcription factors from single hybridization experiments at a reduced cost, using informatics tools such as MAP.

### Comparison with previous p53 and p73 ChIP-based studies

Of note, the ChIP-chip experiments here did not uncover a number of previously known, biologically relevant p53 or p73 targets such as p21Cip1 and Mdm2, because the p53 binding sites in these genes lie outside the 1.5-kb region covered by the NimbleGen arrays. We thus limited the comparison to our results and the previous ChIP-based studies within the 1.5-kb promoter region. A previous chromatin immunoprecipitation and paired-end ditag (ChIP-PET) analysis by Wei *et al*. [7] has identified 541 high-confidence p53 binding sites in 5-flurouracil-treated HCT116 cells, of which 51 are located within the promoter-proximal region covered by the NimbleGen promoter array used in our study and were included in the MAP analysis. Out of these 51 sites, three and four sites showed FDR_MAP _< 0.01 in our p53 (-HU) and p53 (+HU) experiments, respectively. In addition, two and seven sites showed significant FDR_MAP _< 0.01 in our p73 (-HU) and p73 (+HU) experiments, respectively (see Additional file 8). In another genome-wide ChIP-chip study by Smeenk *et al*. [13], 1546 p53 binding sites were identified in actinomycin D-treated U2OS cells. Among those, 39 are located within the region covered by the NimbleGen promoter array used in this study and were included in the MAP analysis. Of these 39 sites, APG-1 and RNASE7 were identified to be bound by p53 in untreated and HU-treated HCT116-3(6) cells, respectively (see Additional file 8). ACTA2, RNASE7, and AXL were identified to be significantly bound by p73 in untreated HCT-116-(3) cells, with FDR_MAP _< 0.005. In HU-treated HCT116-3(6) cells, EPA2R, TRIM22, ACTA2, and RNASE7 were identified to be significantly bound by p73, with FDR_MAP _< 0.005 (see Additional file 8).

We also conducted a comparison between the results from Wei *et al*. [7] and Smeenk *et al*. [13] to evaluate the overlap between their studies. Only about 10% (4 out of 51) sites identified from Smeenk *et al*. [13] overlap with the 54 sites identified from Wei *et al*. [7], suggesting a limited overlap between different studies. Differences in the experimental conditions (i.e. cells, antibody, drug treatment) and methodologies (ChIP-chip versus ChIP-PET) between the studies may account for the limited overlaps. Nevertheless, the various studies have identified promoter targets for p53 and p73. Furthermore, our experimental approach allows for comparisons of the effect of drugs on the promoter occupancy profiles of p53 and p73.

### Promoter binding specificity of p53 and p73

With a focused analysis of p53 and p73 binding sites within promoter-proximal regions, we have found that approximately 50% of the p53-associated promoters contain a p53 consensus site [30], whereas only 20% of the p73-associated promoters contain such a consensus sequence (see Additional file 9). It is possible that p73 may bind to an as yet unidentified non-canonical consensus sequence(s). Alternatively, the promoter association of p73 may be mediated through protein-protein interactions rather than a direct p73-DNA interaction. We have conducted a motif search against the JASPAR transcription factor database and found that p53 and p73-bound promoters are statistically enriched with sequence motifs for the C2H2 zinc finger proteins (i.e. Sp1 and Snail) as well as the basic helix-loop-helix transcription factors (see Figure S3b in Additional file 1). In line with this finding, previous studies have shown that p53 and sp1 can be physically associated and function cooperatively to regulate gene expression of p21Cip1 [31] and Bax [32]. p73 can also interact with sp1 to mediate repression of cyclin B1 transcription [33]. Together with the motif search, our results suggested that the differential promoter occupancy profiles of p53 and p73 in HU-treated cells may be, in part, due to secondary interactions with other transcription factors. Other mechanisms such as post-translational modification(s) of p53 and p73 and/or histone acetylation pattern of the target promoters [34] can also account for the differential effects of HU on the promoter occupancy profiles of p53 and p73.

### p53 and p73 promoter occupancy and regulation of gene expression

It is typically assumed that transcription factor binding and gene expression are correlated. However, several studies have suggested that p53 and p63 binding to the promoters does not directly result in the regulation of transcription [7,8]. In the case of p53, a ChIP-PET study by Wei *et al*. [7] showed that, following 5-fluorouracil treatment, p53 was bound to 474 promoters but only 122 showed changes in gene expression. Espinosa [35] and colleagues have proposed that p53 binding is not the rate-limiting step in promoter regulation, rather it is the post-binding events such as p53 post-translational modifications, chromatin modification and/or recruitment of other transcriptional regulatory proteins that would determine the activity of p53-bound promoters. Thus, binding of p53 to the target promoter is not sufficient to result in transcription regulation. This conclusion is also supported by a ChIP-chip study of p63, by which only 10% to 20% of the p63-bound sites identified by ChIP-chip with FDR < 0.05 showed p63-dependent transcription regulation [8].

### Implications for the phenotypes of the p53 and p73 mouse models

Despite a high degree of similarity among the p53-family members, mouse knockout models suggest that p53 and p73 have differential biological functions and that they are not functionally redundant. The constitutional knockout of p53 does not cause embryonic or neonatal lethality. The most significant phenotype of the p53-null mice is the development of early onset tumors in the testis and thymus that kill the p53-null mice within the first 6 months of life [2,36]. On the other hand, the constitutional knockout of p73 interferes with embryonic development such that the p73-null mice are runt and have defects in neuronal development and immune function, as well as abnormalities in pheromone sensory pathways. The majority of them die of chronic infection by 4 to 6 weeks of age [4]. Recently, Tomasini *et al*. [37] showed that TAp73 isoform-specific knockout mice develop a phenotype intermediate between the phenotypes of p53^-/- ^and p73^-/- ^mice with respect to infertility and spontaneous tumor formation. Moreover, the types of tumor developed in TAp73^-/- ^mice are different from those displayed by p53^-/- ^knockout mice. Taken together, differences in the phenotypes of the p53 and p73 knockout mice suggest that these two related transcription factors have different biological functions, particularly during embryonic development.

In this study, we examined the promoter-binding profiles of p53 and p73 at steady state and under conditions of replication arrest induced by HU. We showed that p53 and p73 are both up-regulated in cells under replication stress. As anticipated, these two related transcription factors indeed bind to a common set of promoters at steady state. Interestingly, however, we found that p73 is recruited to promoters that are not bound by p53 under conditions of replication stress. This finding could explain the different phenotypes of the p53-null and p73-null mice, that is, the p73 protein is involved in the regulation of genes that are not controlled by p53 and those gene functions are required for the proper embryonic development of the mouse.

## Conclusion

Using ChIP-chip in combination with MAP analysis, we performed a direct comparison of p53 and p73 promoter occupancy profiles and our results indicated that p53 and p73 have overlapping and distinct promoter occupancy profiles before and after HU treatment. In addition, HU alters the promoter occupancy profile of p73 more than that of p53. We also found that HU induces the binding of p73 to the MLH3 promoter and causing its up-regulation. This study demonstrates the power of bioinformatics approach to the analyses of promoter occupancy profiles of highly homologous transcription factors. Our data suggest that p73 regulates a different set of genes than p53 in response to genotoxic stress, consistent with the mouse genetic studies showing that these two related transcription factors have different biological functions.

## Methods

### Cell culture and drug treatment

HCT116-3(6) human colorectal cancer cells (ATCC) were grown in high-glucose (4.5 g/liter) Dulbecco's modified Eagle medium (CellGro, Mediatech, Manassas, VA, USA) supplemented with penicillin G (100 U/ml), streptomycin (100 μg/ml), 10% fetal bovine serum (Hyclone, Logan, Utah, USA). Cells at around 80% confluency were treated with 1 mM HU (Sigma) for 16 hours.

### Immunoblotting

Cells were washed with phosphate buffered saline (PBS) and lysed in radioimmunoprecipitation buffer (50 mM Tris, pH 7.4, 150 mM NaCl, 1% Triton X-100, 0.5% deoxycholate, 0.1% SDS, protease inhibitor cocktail) for 15 minutes at 4°C. Lysates were clarified by centrifugation for 15 minutes at 14,000 rpm and supernatants were collected. Protein concentration in the soluble fraction was determined by BioRad DC protein assay. The following antibodies were used at the indicated dilutions to detect the endogenous proteins: mouse monoclonal anti-p53 (DO1; CalBiochem, San Diego, CA, USA) at 1:1000, mouse monoclonal anti-p73 (429; Imgenex, San Diego, CA, USA) at 1:200, and mouse monoclonal anti-p63 (4A4; Pharmingen, San Diego, CA, USA) at 1:300, and mouse monoclonal anti- α-tubulin (Clone B-5-1-2; Sigma) at 1:1000 was used as a loading control.

### ChIP

HCT116-3(6) cells were cultured in 10-cm plates to 70% to 80% confluence prior to drug treatment. Cells were fixed with 1% formaldehyde in serum-free media for 10 minutes at room temperature. Formaldehyde cross-linking was quenched with 125 μM glycine (final concentration). Cells were washed in PBS and nuclei were prepared as previously described [38]. Cell suspension was sonicated 20 seconds for eight times to yield DNA fragments with average length of about 500 bp using Branson 450 sonifier, setting 4 (25% power output). Lysates were pre-cleared with Protein A/G sepharose (GE Healthcare Life Sciences) for 2 hours at 4°C. Approximately 3 × 10^7 ^cells were prepared per immunoprecipitation and incubated with 5 μg of total monoclonal anti-p53 (1:1 mixtures of Ab-1 and Ab-12, CalBiochem) or affinity-purified polyclonal anit-p73 (827) at 4°C overnight. Equivalent amounts of normal IgG were used as negative controls. Protein A/G beads were then added to the immunoprecipitate and incubated for 2 hours. Beads were then washed sequentially once in low salt buffer, once in high salt buffer, and three times in TE. DNA/protein complexes were eluted and decross-linked by heating at 65°C overnight [38,39]. Eluates were treated with Proteinase K and DNA fragments were extracted with phenol/chloroform twice and treated with RNase A. DNA fragments were then purified and eluted in TE using QIAquick PCR purification columns (Qiagen).

### NimbleGen promoter array analysis

Total input and ChIP DNA prepared from untreated or HU-treated cells were amplified by LM-PCR as previously described [40] and hybridized to 1.5-kb promoter arrays manufactured by NimbleGen Systems Inc. (Madison, WI, USA). The 1.5-kb promoter array platform is a single-array design that covers 24,135 human promoters. For each promoter, 15 50-mer probes were designed to tile across the proximal region from -1300 to +200 relative to the transcription start site. Probe labeling and hybridization were performed by NimbleGen (Reykjavik, Iceland).

### Quantitative PCR analysis

Selected promoters were verified by SYBR Green quantitative PCR analysis. Primers were designed using Primer Express v2.0 (Applied Biosystems, Foster City, CA, USA). Each PCR reaction was performed in duplicate in a 25 μl reaction with 5 ng of LM-PCR amplified input or ChIP DNA and 1× Power SYBR Green master mix (Applied Biosystems). Fluorescence values were determined by ABI Prism 7900 HT sequence detection system. Fold enrichment was calculated in terms of occupancy units as described in Yang *et al*. [8]. Fold enrichment (OU) = 1.9^-(delta CT^_expt_^-delta CT^_Tk1_) where delta CT = CT_IP_-CT_Input_. Specificity of the PCR reaction was confirmed by the presence of a single peak in dissociation curve analysis and a single product on agarose gel. Primer sequences are listed in Additional file 10.

### Data analysis for NimbleGen promoter arrays

A MAP algorithm was developed based on the MAT algorithm [17] to identify p53 and p73 ChIP-enriched sites. Details are described in Additional file 2. ChIP-chip datasets have been deposited in the Gene Expression Omnibus (GEO) database under the accession number GSE12224.

### siRNA transfection

siRNA duplexes against p53, p73, and lacZ were obtained from Dharmacon (Lafayette, CO, USA) as described in Chau *et al*. [39]. HCT116-3(6) cells were transfected in suspension with RNA duplexes using Oligofectamine (Invitrogen).

### shRNA and lentiviral infection

We used p73 MISSION shRNA (NM_005427) (Sigma) to establish stable p73 knockdown cell lines from HCT116-3(6). pRS-lacZ and pRS-p53, kindly provided by Brummelkamp *et al*. [41], were used to establish stable p53 knockdown cell lines from HCT116-3(6) cells. HCT116-3(6) cells were transduced with viral supernatants containing p73 shRNA or p53 shRNA collected from 293 amphotropic packing cell lines 48 hours following transfection using a standard virus transduction protocol. Infected cells were selected for puromycin resistance and were re-seeded at low density to obtain single colonies. Positive clones were confirmed by Western blotting analysis.

### Gene expression analysis

Total RNA from HCT116-3(6) cells transiently transfected with lacZ or p53/p73 siRNA oligos with/without treatment of HU was isolated using RNeasy Mini Kit (Qiagen), according to the manufacturer's instructions. Total RNA was converted to cRNA, labeled with Cy5 or Cy3 dUTP using Agilent low input linear RNA amplification, and the resulting cRNA was hybridized to the Phalanx Human One array. After hybridization, the slides were scanned with Axon 4000 B, and the raw data were generated by Genepix 6.0. Four biological repeats were performed for each condition (-HU or +HU), with two technical repeats (dye swap) per biological sample. The data were normalized with quantile normalization of DNAMR [42] and fitted with the linear model from the R package Limma [43]. Two comparisons were performed: Gene expression in lacZ siRNA-transfected cells versus p53 and p73 siRNA-transfected cells in the -HU or +HU condition. *P *value was calculated using *t*-statistics. To integrate ChIP-chip data with gene expression profiling, we first selected genes common to the two array platforms. We then selected promoters from the four experiments using FDR_MAP _< 0.05. Promoters whose expression was affected by p53/p73 knockdown are shown (*P *< 0.1). Raw data have been deposited in the GEO database under the accession number GSE12242.

### RNA extraction and real-time RT-PCR analysis

Total RNA was extracted by using RNeasy Mini Kit (Qiagen), according to the manufacturer's instructions. Total RNA (500 ng to 1 μg) was reverse transcribed by using the High-Capacity cDNA Archive Kit (Applied Biosystems). The resulting cDNA samples were subjected to RT-PCR analysis using the following gene-specific primers. p21cip1: GCAGACCAGCATGACAGATTTCT (fwd), GCGGATTAGGGCTTCCTCTT (rev). MLH3: CCTGCTTGAGGGCTGCAT (fwd), CTCAAATTGGTCTGGCCTTAAAA (rev). ETF1: CCAAGGAGCATACCCATGGT (fwd), AAGTTCTGGAAATGTTCCAATTGTAA(rev), ACTB: CGAGAAGATGACCCAGATCATGTT (fwd), CCTCGTAGATGGGCACAGTGT (rev).

### GO term analysis

Functional classifications of target genes were performed using the web-based program Database for Annotation, Visualization, and Integrated Discovery (DAVID) [44]. Each identified category is provided by a *P *value of the likelihood of finding the identified category by random chance. Categories represented >5% with *P *< 0.01 were selected.

### Motif search

Using the cut-off of FDR_MAP _< 0.005, we extracted the 2.5-kb query sequence (1.5-kb probe region plus 500 bp surrounding either side of the 1.5-kb probe region in each promoter) from the human chromosome build 35 (hg17) assembly. We used p53scan [13,45] to search for p53 motifs in p53- and p73-bound promoters. We also searched p53- and p73-bound promoters against the JASPAR database [46] using the motif search program Cis-eLement OVERrepresentation [47] for other over-represented motifs corresponding to 123 curated transcription factor-binding motifs using *P *value cutoff < 0.001.

### Correlation analysis

Correlation between ChIP-chip experiments is determined by calculating the Pearson correlation coefficient (r) derived from linear regression analysis. *P *values were calculated to test the significance of the correlation. The test statistic is based on Pearson's product moment correlation coefficient cor(x, y) and a t distribution with length(x)-2 degrees of freedom, assuming the samples follow independent normal distributions.

## Abbreviations

ChIP: chromatin immunoprecipitation; ChIP-chip: chromatin immunoprecipitation on DNA chip; ChIP-PET: chromatin immunoprecipitation and paired-end ditag; FDR: false discovery rate; GEO: Gene Expression Omnibus database; GO: gene ontology; HU: hydroxyurea; IgG: immunoglobulin G; LM-PCR: ligation-mediated PCR; MAP: model-based algorithm for promoter array; MAT: model-based algorithm for tiling array; MSCV: murine stem cell virus; OU: occupancy unit; PBS: phosphate buffered saline; PCR: polymerase chain reaction; qChIP: chromatin immunoprecipitation and quantitative PCR analysis; RT-PCR: reverse transcription PCR; shRNA:small hairpin RNA; siRNA: small interfering RNA.

## Authors' contributions

VH performed ChIP-chip, validation experiments, and gene expression profiling experiments to identify p53 and p73 regulated promoters. XL developed the MAP algorithm and provided the bioinformatics support for the analyses of the ChIP-chip data. YJ assisted in gene expression profiling experiments and performed the motif analysis. VH and JYJW wrote the paper.

## Supplementary Material

Additional file 1 **Supplementary figures**. Figure S1 – Confirmation of antibody specificity used in this study. Figure S2 – Verification of model-based algorithm for promoter array window and promoter-scores. Figure S3 – Motif analysis. Click here for file

Additional file 2 **Supplemental document**. A detailed description of model-based algorithm for promoter array used for NimbleGen data analysis. Click here for file

Additional file 3 **Table S1**. Table S1 contains false discovery rate and model-based algorithm for promoter array (MAP) scores from the MAP analysis for the four chromatin immunoprecipitation on DNA chip experiments: p53(-HU), p53(+HU), p73 (-HU), and p73 (+HU); HU = hydroxyurea. The same analysis was performed for OCT4 and immunoglobulin G as negative controls. Click here for file

Additional file 4 **Table S2**. Table S2 contains a list of model-based algorithm for promoter array selected promoters using FDR_MAP _< 0.005. Click here for file

Additional file 5 **Table S3**. Table S3 is a list of promoters whose expression was significantly affected by p53 and p73 double knockdown in untreated or hydroxyurea-treated cells. Click here for file

Additional file 6 **Table S4**. Table S4 is a list of promoters bound by p73 only in hydroxyurea-treated cells with FDR_MAP _< 0.005. Click here for file

Additional file 7 **Table S5**. Table S5 is a summary of the gene ontology term analysis of the p53 and p73 bound promoter in untreated and hydroxyurea-treated HCT116-3(6) cells. Click here for file

Additional file 8 **Table S6**. Table S6 is a comparison between the p53 and p73 binding sites identified in our study with previous chromatin immunoprecipitation-based studies. Click here for file

Additional file 9 **Table S7**. Table S7 is a summary of p53 motif search using p53scan. Click here for file

Additional file 10 **Table S8**. Table S8 is a list of primer sequences used for quantitative chromatin immunoprecipitation experiments. Click here for file
