# Supplementary material for: Effect of hydroxyurea on the promoter occupancy profiles of tumor suppressor p53 and p73
Source: BMC Biol. 2009 Jun 26;7:35. doi: 10.1186/1741-7007-7-35 (PMC2711048; doi:10.1186/1741-7007-7-35)
Supplement: Additional file 1 — Supplementary figures. Figure S1 – Confirmation of antibody specificity used in this study. Figure S2 – Verification of model-based algorithm for promoter array window and promoter-scores. Figure S3 – Motif analysis. [file 1741-7007-7-35-S1.pdf]

Figure S1

A

HCT116-3(6)	p53	p63	p73
β-actin normalized CT value	20.23 ±0.005	30.33 ±0.66	28.18 ±0.11

B

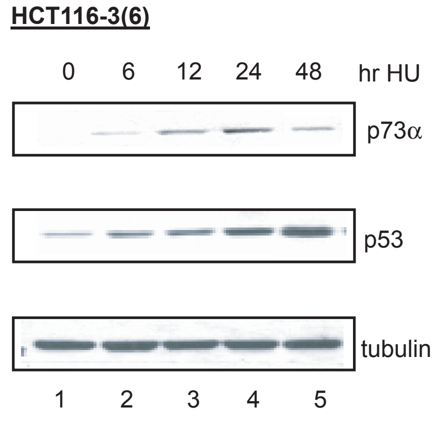

C

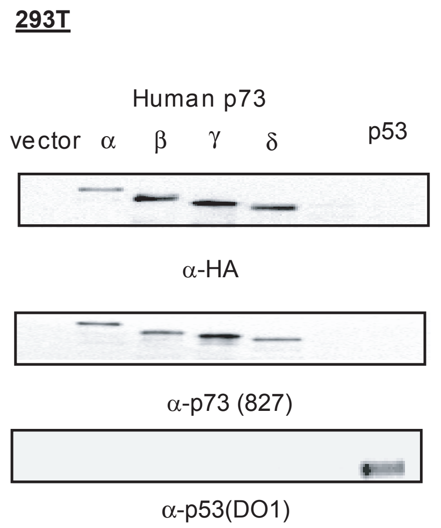

Figure S1 - Confirmation of antibody specificity used in this study.

(a) Relative expression of p53, p63, and p73 in HCT-116(3) cells. mRNA level was determined by real-time polymerase chain reaction shown as β-actin normalized Ct values. Error represents standard deviation from three biological samples. **(b)** Time-course of p53 and p73 induction by hydroxyurea. HCT116-3(6) cells were treated with 1 mM hydroxyurea at the indicated times. Endogenous levels of p53 and p73 were detected by immunoblotting as described in Figure 1(a). **(c)** Validation of p53 and p73 antibodies used in this study. Extracts of 293T cells transfected with empty vector, HA-tagged p53, or HA-tagged p73 α/β/χ/δ were subjected to SDS-PAGE followed by immunoblotting with the indicated antibodies.

Figure S2

A

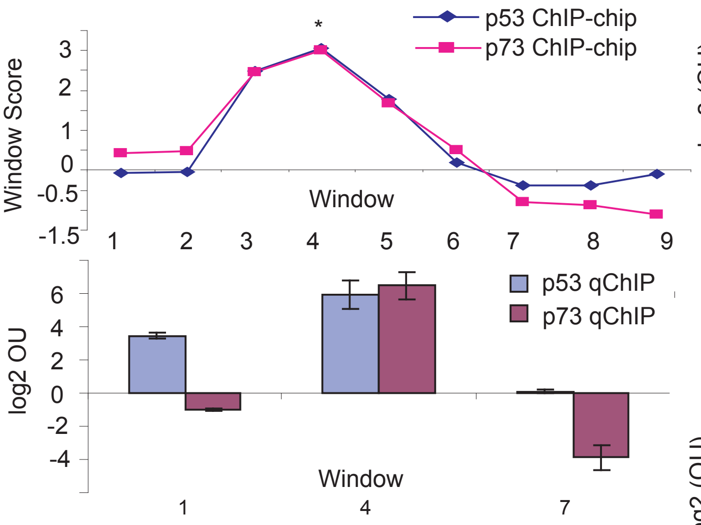

Chromosome X position

1: 77389053-77389233 (180 nt, p=3)
2: 77389403-77389603 (200 nt, p=3)
3: 77389603-77389823 (220 nt, p=3)
4: 77389753-77389883 (130 nt, p=3)
5: 77389823-77390053 (230 nt, p=3)
6: 77389883-77390103 (220 nt, p=3)
7: 77390053-77390273 (220 nt, p=4)
8: 77390103-77390333 (230 nt, p=4)
9: 77390273-77390483 (210 nt, p=3)

B

qChIP verification scheme

Select 20 promoters with FDR <0.05 in the IP channels and FDR >0.8 in the input channels and with similar promoter scores

↓

Design primers to amplify the region defined by the promoter score

↓ p53 and p73 ChIP qPCR

Calculate Occupancy Unit (OU)
log 2 (OU)>1: positive confirmation

C

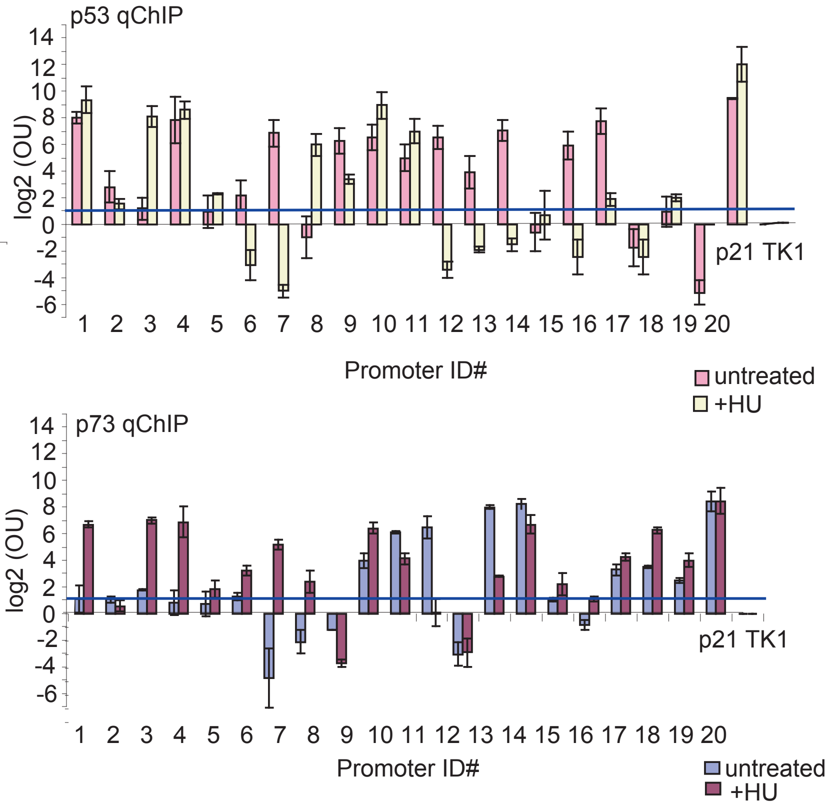

D

p53 -HU

MAP Computed FDR	Promoter Score Range	# false/# assayed	qChIP FDR	# promoters in bin
0-0.005	1.22-2.15	2/12	0.17	**201**
0.005-0.01	1.11-1.14	1/3	0.33	65
0.01-0.05	0.96-0.99	3/5	0.60	183

p53 +HU

MAP Computed FDR	Promoter Score Range	# false/# assayed	qChIP FDR	# promoters in bin
0-0.005	1.14-2.03	6/16	0.37	**216**
0.01-0.05	0.98-1.01	2/4	0.50	173

p73 -HU

MAP Computed FDR	Promoter Score Range	# false/# assayed	qChIP FDR	# promoters in bin
0-0.005	1.08-2.41	7/19	0.37	**360**

p73 +HU

MAP Computed FDR	Promoter Score Range	# false/# assayed	qChIP FDR	# promoters in bin
0-0.005	0.89-2.13	4/19	0.21	**526**

Figure S2. Verification of model-based algorithm for promoter array window and promoter-scores.

(a) Window score verification. Quantitative chromatin immunoprecipitation (qChIP) scanning of p53 and p73 across a representative promoter region. Window scores across the indicated chromosome regions (top) and the enrichment of selected chromosome regions determined by qChIP (bottom) in a logarithmic-scale (log2) are shown. *denotes genomic region that contains a non-canonical p53 consensus sequence: GGGCTTGCTg (spacer = 32)AGGCAAGTgT (capital letters represent perfect match to the consensus sequence), p = number of probes. **(b)** Verification strategy. **(c)** Promoter-score verification. Summary of p53 and p73 qChIP verification of the 20 selected promoters in the untreated hydroxyurea (-HU) or hydroxyurea-treated (+HU) experiments. Two independent ChIPs were performed for the –HU or +HU condition using the indicated antibodies. Input and ChIP DNA were amplified by ligation-mediated polymerase chain reaction. Enrichment was expressed in terms of log2 occupancy unit (OU). p21cip1 and TK1 were used as controls for the calculation. Data are represented as mean of the log2 OU from two independent ChIPs +/- standard error of the mean. Log2 (OU) > 1 is considered as a positive confirmation (indicated by the blue line). **(d)** Summary table of model-based algorithm for promoter array analysis, qChIP verification, and false discovery rate determination.

Figure S3

A.

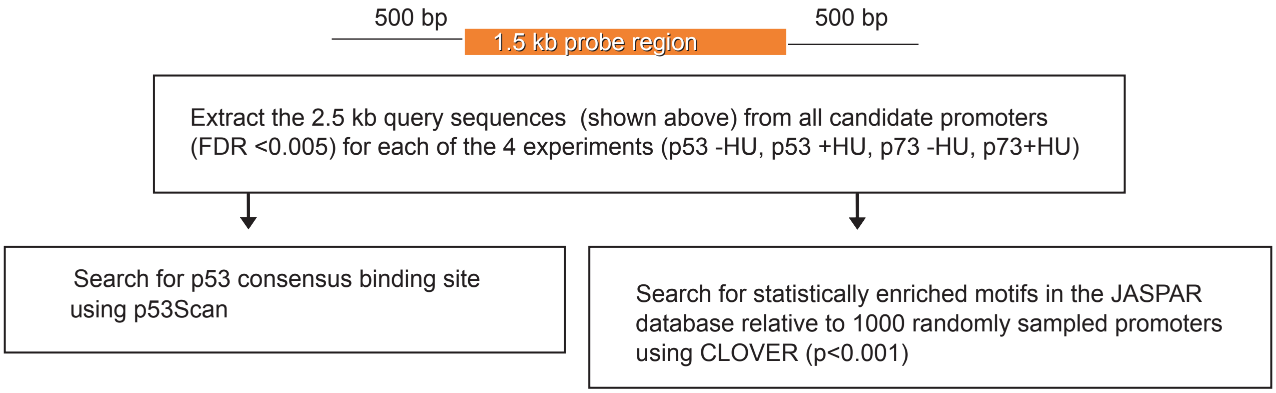

B.

p<0.001				Experiment			
JASPAR Motif ID	Motif name	Motif type	Motif Logo	p53 -HU	p53+HU	p73 -HU	p73 +HU
MA0010	Broad-complex_1	C2H2 ZN-FINGER	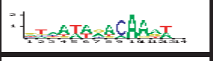				X
MA0013	Broad-complex_4	C2H2 ZN-FINGER	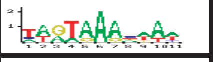	X	X	X	X
MA0039	Gklf	C2H2 ZN-FINGER	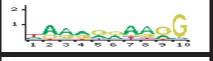			X	
MA0041	HFH-2	FORKHEAD	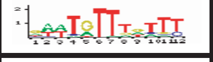			X	X
MA0045	HMG-IY	HMG	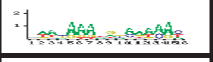			X	X
MA0049	Hunchback	C2H2 ZN-FINGER	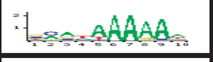	X	X	X	X
MA0055	Myf	bHLH	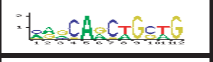		X	X	X
MA0056	MZF_1-4	C2H2 ZN-FINGER	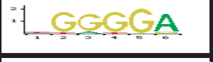	X	X	X	X
MA0068	Pax-4	PAIRED-HOMEO	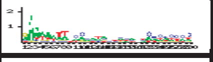			X	X
MA0073	RREB-1	C2H2 ZN-FINGER	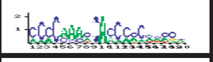	X	X	X	
MA0079	SP1	C2H2 ZN-FINGER	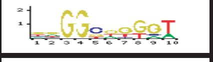	X	X	X	X
MA0082	SQUA	MADS	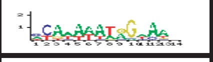	X		X	X
MA0086	Snail	C2H2 ZN-FINGER	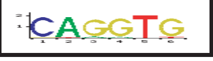	X	X	X	X

Figure S3. Motif analysis.

(a) Outline of motif search for the p53- and p73-bound promoters. **(b)** Over-represented JASPAR motifs in p53 and p73-bound promoters. The sequence logo of the JASPAR motif represents the frequency of occurrence in the binding-site matrix. A motif that is over-represented with *P* < 0.001 in the experimental set is marked by X. *P* values are calculated to indicate the degree of over-representation of each PSSM in target versus background sequences randomly selected from 1000 human promoters. Motifs found in all four experiments are highlighted in yellow.
