# Supplementary material for: Effect of hydroxyurea on the promoter occupancy profiles of tumor suppressor p53 and p73
Source: BMC Biol. 2009 Jun 26;7:35. doi: 10.1186/1741-7007-7-35 (PMC2711048; doi:10.1186/1741-7007-7-35)
Supplement: Additional file 2 — Supplemental document. A detailed description of model-based algorithm for promoter array used for NimbleGen data analysis. [file 1741-7007-7-35-S2.doc]

**Model-based Analysis of Promoter Arrays (MAP)**

The probe-level hybridization signals were processed by a method similar to the Model-based Analysis of Tiling-arrays (MAT) proposed by Johnson et al. (Johnson et al., 2006). The MAP algorithm relies on the assumption that in a ChIP-chip experiment only a very small fraction of the probes will hybridize to the protein-bound DNA sequences, while the majority of the probes will measure only background noise, which can be influenced by the sequence configurations of the probes. Therefore, we can model the actual binding sites using a sequence specific model that relates the probes signals to their sequence characteristics. In other words, MAP can greatly reduce the background noise caused by probe copy number effect and probe sequence characteristics (i.e. GC content). Instead of comparing the signals of individual probes across a very limited number of experimental repeats, we can borrow information from thousands of probes on the chip with similar sequence configurations to eliminate sequence specific noises, and to achieve a much better result in locating true binding sites.

First, the 50-mer probes used were mapped onto build 36.1 finished human genome assembly (hg18, Mar 2006) downloaded from the UCSC Genome Bioinformatics Site (<http://genome.ucsc.edu/>) using the extreme MApping of OligoNucleotides (xMAN) software developed by Shirley Liu’s group (<http://chip.dfci.harvard.edu/~wli/xMAN/>). Out of the 353,126 probes on the NimbleGen arrays, 1,214 (0.34%) of them could not be mapped due to the update of human genome sequences, 345,061 (97.72%) of them were mapped to exactly 1 location, 351,815 (99.63%) of them had 1 to 9 mapped locations and 97 (0.03%) of them had 10 or more mapped locations. Only probes having 1 to 9 mapped locations were used in the following model based analysis.

Following probe filtering based on mapped locations, the log of probe signals in each channel of the experiment were fitted by a linear model (Johnson et al., 2006):

$$\log(y_{i})=\alpha n_{iT}+\sum_{j=1}^{50} \sum_{k\in\{A,C,G\}} \beta_{jk}I_{ijk}+\sum_{k\in\{A,C,G,T\}} \gamma_{k}n_{ik}^{2}+\delta\log(c_{i})+\varepsilon_{i}$$

where

- *y_i_* is the signal level of the *ith* probe, *i* = 1, …, 351,815;
- *n_ik_* is the count of nucleotide *k* in the *ith* probe, *k* ∈ (Barrera and Ren, 2006 G);
- *α* is the baseline value (intercept or constant) based on the number of nucleotide T in the probe, e.g., 50*α* is the baseline when the probe sequence is a run of 50 Ts;
- *I_ijk_* is an indicator function, *I_ijk_* = 1 if the nucleotide at position *j* is *k* in the *ith* probe, and *I_ijk_* = 0 otherwise;
- *β_jk_* is the effect of each nucleotide *k* (except T, which is already modeled in *α*) at position *j*;
- *γ_k_* is the effect of nucleotide count squared;
- *δ* is the effect of the log of the probe copy number;
- *c_i_* is the number of times that the sequence of the *ith* probe appears in the genome by mapping the 50-mer probe sequence against whole human genome sequence;
- *ε_i_* is the probe-specific error term, assumed to follow a Gaussian distribution.

There are 156 parameters in this linear model (1 for *α*, 3*50 for *β_jk_*, 4 for *γ_k_*, and 1 for *δ*). For each channel, the probe signals were fitted by the above model and the 156 parameters were estimated using robust linear regression (Venables, W. N. and Ripley, B. D. (2002) Modern Applied Statistics with S, 4th edition. Springer). We used a robust linear regression instead of the simple least square regression proposed by Johnson et al. (Johnson et al., 2006). With robust linear regression, outliers will be down-weighted so the model can better approximate the background signals.

After estimating the sequence specific parameters, we calculated the model fitting residues for each probe on the chip and used the model fitting residues as the standardized probe fluorescence values. According to NimbleGen’s chip design files, the probes were grouped into 24,092 promoter regions with most of the regions having 15 probes. In each promoter region, we used a sliding window of 250 bases to calculate window scores as the median of the probe fluorescence values in a window containing at least 3 probes. We then assigned the maximum window score as the score for the promoter. All window scores were recorded to control for the rate of false discovery. When there is no specific protein binding DNA hybridized on the chip, the model fitting residues as well as the window scores will follow a Gaussian distribution, which is symmetric to 0. Therefore, the negative tail of the pooled window scores can be used to approximate the NULL distribution and control for false discovery within each channel. For each promoter score *x*, we first calculated

$$F(x)=\frac{\sum_{i} I_{w_{i}>x}/nW}{\sum_{j} I_{p_{j}>x}/nP}$$

Where *i* goes through all windows, *wi* is the *ith* window score, *nW* is the total number of windows we calculated, *j* goes through all promoters, *pj* is *jth* promoter score, *nP* is the total number of promoters, $I_{w_{i}>x}$and $I_{p_{j}>x}$are indicator functions. Then the False Discovery Rate of *x* is calculated in a way similar to that of *q*-value proposed by Storey et al 2003 (Storey and Tibshirani, 2003) to guarantee a monotonic relationship between the promoter score and their FDR.

$$FDR(x)=\min(F(t):t<=x)$$

We noticed that although the variations of the standardized probe fluorescence values are very close in all four IP and input channels, the promoter scores calculated in the input channels have much higher variation than the IP channels. This may due to the fact that in input channels the DNA were randomly amplified and the amplification bias introduced additional variation to the calculation of promoter scores. Therefore, we could not use the promoter scores from input channels to control the FDR for IP channels, even after standardized the standard deviations. We also tried to subtract window scores of the input channel from corresponding IP channel, but the result was not better than controlling FDR within individual channels.

With different FDR cutoffs, we identified 500-3000 promoters from the 4 IP channels, and a relatively smaller number of false positive sites from the input channels. As a negative control, we also performed MAP analysis on the IgG channels from a study by Xu et al. (Xu et al., 2007).

**Table 1. Summary of MAP analysis**

| FDR cutoff | p53 untreated IP | p53 untreated Input | p53  HU  IP | p53  HU  Input | p73 untreated IP | p73 untreated  Input | p73  HU  IP | p73  HU  Input |
| --- | --- | --- | --- | --- | --- | --- | --- | --- |
| 0.001 | 575 | 211 | 615 | 81 | 1006 | 279 | 1407 | 152 |
| 0.005 | 1083 | 374 | 1062 | 84 | 1359 | 489 | 1889 | 183 |
| 0.01 | 1310 | 687 | 1199 | 136 | 1555 | 787 | 2129 | 268 |
| 0.05 | 1873 | 1464 | 1778 | 750 | 2210 | 1718 | 2642 | 985 |
