# Supplementary material for: Effect of hydroxyurea on the promoter occupancy profiles of tumor suppressor p53 and p73
Source: BMC Biol. 2009 Jun 26;7:35. doi: 10.1186/1741-7007-7-35 (PMC2711048; doi:10.1186/1741-7007-7-35)
Supplement: Additional file 10 — Table S8. Table S8 is a list of primer sequences used for quantitative chromatin immunoprecipitation experiments. [file 1741-7007-7-35-S10.pdf]

Table S8

A

NimbleGen Seq ID	Gene Acession ID	Val. ID	Forward	Reverse	Verification on gel
HSAP0406S00027218	AJ223280	1	TCTTGCACCCCGTCTTCAC	CCAGAGCCCTGAGGAATGAC	Yes
HSAP0406S00021203	NM_000040	2	GCCTGCCTGGATTGAAACC	GCACAGAAGACCAGGCATCA	Yes
HSAP0406S00024903	AB007865	3	AGTCATCACCTCTACCTTGAAACTAGTCT	ACACTGCTGAATCCACTCAATCA	Yes
HSAP0406S00001733	AY129015	4	GCCAGAGGTTTAAAAAGGTTAAGATTT	GCGGCAGGACAACATGGT	Yes
HSAP0406S00032402	NM_032488	5	AGACCAGCCTGGGCAACA	CCCGGCTAATTCTATTTGTATTTTTT	Yes
HSAP0406S00002422	M26316	6	AGAAAGACCACGAAGACCACCTAA	GCTGCTCCTCACAGTGCTTACA	Yes
HSAP0406S00003249	NM_014002	7	CCCAGCTGCCCATCAAGAT	GCCACAGAGAAGCCAGGAACT	Yes
HSAP0406S00019175	NM_014661	8	CACACCGGACCCATTCTTTT	GAGCGAGCTGTAGTGTGAAAGGA	Yes
HSAP0406S00036295	AL831883	9	TCTGGGTCTGACATCAACCATTT	TGTTTCAGAAAGATAGGAGATAGCTCTGT	Yes
HSAP0406S00034186	AK127768	10	GCTTACAACTGGTGTGTGTCTTAGG	TGCTCTCAAAGGCGGAACA	Yes
HSAP0406S00034239	NM_005286	11	CCAGTGCTTCGAGTCCAGAAG	GACTGCCTGTACTGGCCAAAA	Yes
HSAP0406S00036196	NM_006639	12	CTGGAATGACTGGGCTTGCT	CTTGCCTGATCCCCCAAAT	Yes
HSAP0406S00027800	AK122592	13	GTAAAGTCCCCCAAACCAAAGTT	CGACTTAGCAACCACCTCATAGG	Yes
HSAP0406S00018051	AK025655	14	ACAGGGAGTGCAGTCTTAAATGG	AAAAAATGCAACCACAGCTATCC	Yes
HSAP0406S00017623	AK124271	15	GCAGCGGTACCTGACCAGAA	CTTCCTGTGCTTCCTACAACTTTTC	Yes
HSAP0406S00008775	BC026264	16	GCTGTAATTCAGCTAATATCAATGATCTTG	TGCTACTCTTATGCACACTATTCTGAAA	Yes
HSAP0406S00016016	AF370371	17	TTGTTCTGTTGGTGCCATGAG	TCATGTCTGGAGGCTGAGGTT	Yes
HSAP0406S00009756	AK124770	18	AGAAGGATAGAAATTGGGTTTGTAGAAT	AAAACTGCGGGCTCTGACA	Yes
HSAP0406S00004567	NM_138938	19	TAGCTCCTCCCTGGGTCTCA	ACCATATCCCACCAGAGAGGTAAG	Yes
HSAP0406S00011489	NM_000410	20	TCCCCTCCCAACTGCTAATG	CCTAACCCCAGACACACTGATTT	Yes
p21cip1	NM_078467	n/a	AGCCTCCCTCCATCCCTATG	TTCTATGCCAGAGCTCAACATGTT	Yes
TK1	NM_003258	n/a	AGTCCCTCCCTGCAATCCA	TGGGTTTCCCAAGCAAGGT	Yes
MLH3	NM_014381	n/a	GGAGTCCCGCTTAACAAATTCTC	ACGAGCCTCAAGATCCAAGGT	Yes
ETF1	BC014269	n/a	CCC CGT CAG GCT CGA AT	AAG CCT CAG ATG TCC ACC CTA TT	Yes

B

NimbleGen Seq ID	Gene ID	Forward	Reverse
HSAP0406S00013662	NM_021223	CAGGGCCCAGCCTCCTT	TCCTCTCCTTATTTGGACCTTCAG
HSAP0406S00031561	NM_005583	TGCCACCCACTTGTGATACTCA	GCTCTTGCAGTTGCCCAGAT
HSAP0406S00025741	AB032525	CGAGGCATGGACCTGAAAAC	TCTTCCTTGGATTCCCTATTGC
HSAP0406S00022455	AK094261	GGCTACTGCTGCTTCAAGACAGT	TCACCAGCGCTCCTGACA
HSAP0406S00034475	NM_058186	ACTGTTCTGGAGGAAGCAAAGAA	CAAGTTCGCCGTCCATGTC
HSAP0406S00016860	NM_006378	AGACCTGCCCCAGGAAGTG	TTTATTGTCCTTTCAGGACACCAA
HSAP0406S00023356	AK127211	ACTTCTCCCCCTTCCAGAACTC	GTCGCCGTAGCAGGTCAGA
HSAP0406S00006063	NM_024080	GAAAGTTGCCCACTGACAATTG	TGTCCCCTTTAAACTACACTTTGGA
HSAP0406S00019480	NM_053017	CTGCAGAGGTCATGGGAGAAG	CTCTGGCTGCTAGGAAGCTCTT
